# Adherence to antiretroviral therapy and determining factors in adults living with HIV receiving services at public health facilities amidst the COVID-19 crisis in Bahir Dar city, Northwest Ethiopia

**DOI:** 10.3389/fpubh.2024.1380055

**Published:** 2024-04-19

**Authors:** Chalachew Dessie Gela, Gebiyaw Wudie Tsegaye, Belayneh Fentahun Shibesh

**Affiliations:** ^1^Department of Epidemiology and Biostatistics, School of Public Health, College of Medicine and Health Science, Bahir Dar University, Bahir Dar, Ethiopia; ^2^HIV/AIDS Prevention Treatment and Care, Amhara Regional State Health Bureau, Bahir Dar, Ethiopia

**Keywords:** adherence, antiretroviral therapy, COVID-19, HIV-positive adult, Ethiopia

## Abstract

**Background:**

The COVID-19 pandemic has presented new challenges to adhering to ART, and its influence on adherence and related factors has not been thoroughly studied. This study examines ART adherence and its associated factors during the COVID-19 pandemic.

**Methods:**

A cross-sectional study was conducted on HIV-positive individuals receiving care and treatment in public health facilities. A total of 612 participants were selected using a multi-stage sampling procedure. Data were collected through interviewer-administered questionnaires and chart reviews. We used Epi-data for data entry and Stata for data analysis.

**Results:**

Good adherence to antiretroviral therapy in this study was 76.5% (95% CI, 72.9, 79.7). Divorced marital status (AOR = 0.45,95%CI:0.22,0.90), regular follow-up (AOR = 3.01,95%CI:1.81,5.01), adherence counseling and information in the context of COVID-19 pandemic (AOR = 2.57,95%CI:1.63,4.08), and knowledge about ART (AOR = 1.81,95%CI:1.11,2.94) were significantly associated with adherence to antiretroviral therapy.

**Conclusion:**

The observed adherence rate in this study was lower than the World Health Organization recommendation and previous studies. The study highlighted the importance of addressing adherence to ART among HIV-positive adults during the COVID-19 pandemic and other upcoming emerging and reemerging outbreaks. Strategies to improve adherence should consider factors such as marital status, regular follow-up, provision of counseling and information, and enhancing knowledge about ART.

## Background

As per the Federal HIV/AIDS Prevention and Control Office (FHAPCO) in Ethiopia, there is an estimated total of 613,000 people living with HIV (PLHIV) in the country. Among them, 30% are reported from the Amara region (1). The World Health Organization (WHO) advises an ART adherence rate of at least 95% to prevent the emergence of drug-resistant strains and transmission. Unfortunately, the reported adherence rates are frequently below this recommended threshold (2).

The emergence of the COVID-19 pandemic has added a health burden to PLHIV (3). In the era of the COVID-19 pandemic, the provision of ART services may be affected due to overwhelmed healthcare systems, interruptions in services, and reduced clinic visits by PLHIV due to fear or financial constraints, resulting in suboptimal adherence to treatment. There is limited available data on PLHIV in the context of the ongoing SARS-CoV-2 pandemic (4). Managing COVID-19 directly impacts the local HIV prevention and care landscape, which heavily relies on digital HIV intervention programs (5). Owing to their immunosuppressed state, PLHIV are presumed to be more susceptible to COVID-19 infection and may experience worse clinical outcomes (6).

Factors such as the adverse effects of certain ARVs, lack of social support, stigma, inadequate knowledge about ART, opportunistic infections, and limited access to healthcare services significantly contribute to challenges in adhering to ART (7–12).

The COVID-19 pandemic disrupted the supply chain of HIV-related products from the global to local levels. This disruption may exacerbate the occurrence of severe illnesses and increase mortality rates (13). Investigating if the COVID-19 pandemic affects adherence to antiretroviral treatment is essential (14).

There is limited understanding of how COVID-19 affects the clinical progression of individuals living with HIV in Ethiopia. Although various studies have explored factors related to adherence to ART, there is a lack of documentation on the impact of COVID-19 pandemic-related factors on ART adherence. Therefore, the primary objective of this study was to evaluate the influence of the COVID-19 pandemic on ART adherence and its associated factors among adults living with HIV who receive care and treatment at public health facilities.

## Methods

### Study designe and setting

An institutional-based cross-sectional study was carried out among adult clients receiving ART follow-ups at public health facilities in Bahir Dar city from November 29 to December 28, 2020. Bahir Dar’s city administration includes three public hospitals and ten public health centers, all offering comprehensive HIV prevention, treatment, and care services. The total count of clients receiving ART in Bahir Dar health facilities was 14,502.

### Data collection and participants

The sample size was determined using EPI info software. Assuming a 95% confidence level, a 5% margin of error, and a proportion of good adherance 56% (15). An additional 10% was added to account for non-response and incomplete follow-up charts. Additionally, a design effect of 1.5 was used, resulting in 626 study participants. Multi-stage sampling was employed to select participants. The study included two hospitals and four health centers, chosen through a simple random (lottery) method from a pool of three hospitals and nine health centers, respectively. The sample size was then allocated proportionally to each selected health facility based on the number of adults currently undergoing ART treatment. Participants were selected using a systematic sampling technique with an interval of every fourth individual. The first participant to arrive at the ART unit for follow-up on each day and at each facility was selected as the initial interviewee. Subsequent participants were selected at every fourth interval based on their order of arrival until the required sample size for each selected health facility was achieved. To assess adherence to ART, good adherence was defined as taking at least 95% [≤ 2 missed drug doses out of 30 or ≤ 3 missed drug doses out of 60 doses (16)] of prescribed medication within one month before data collection (2, 17). Adequate knowledge was determined based on respondents correctly answering questions that assess their knowledge at or above the mean level (12). Problematic alcohol use was identified if the respondent scored ≥2 on the CAGE tool (18). A client is considered to have experienced a side effect if they encounter any adverse medical event during their treatment with a pharmaceutical product, which is duly documented in their patient chart. A client is classified as having a history of hospitalization if there is documented evidence of any prior hospital admissions related to HIV in their medical records. Clients categorized as having regular ART follow-up adhere to their scheduled appointments for ART refills, as indicated by their attendance based on the predetermined schedule outlined in their patient follow-up chart, either as scheduled or unscheduled visits recorded by the care provider. A client is considered to have been exposed to stigma if they encounter any from of adverse attitudes, beliefs, behaviors, or actions directed toward them or their community due to their HIV/AIDS status.

### Inclusion and exclusion criteria

The study included all HIV-positive adults aged 18 or older receiving follow-up care at public health facilities, excluding those receiving treatment for less than a month.

### Survey instrument and data quality assurance

Data collection involved the utilization of structured questionnaires administered by the interviewer, and additional information was extracted from client charts as secondary data. The questionnaire was prepared by selecting, modifying, and adapting relevant evaluation tools from previous studies conducted in English. Dose adherence was calculated by subtracting missed doses from prescribed doses, dividing the result by prescribed doses, and multiplying by 100 to determine the outcome variable (2, 17). Different measures were implemented to ensure high-quality data, such as conducting a pretest on 5% of participants, providing extensive training to data collectors, translating the questionnaire from English to Amharic (the local language), and back-translating it into English to ensure consistency. The principal investigator was responsible for supervising the entire data collection process.

### Statistical analysis

The collected data were coded, cleaned, and entered into EpiData version 4.6 before being exported to Stata version 14 for processing and analysis. Descriptive statistics were used to describe respondent characteristics. Frequency, proportion, and summary statistics were computed and reported in tables. The bivariate analysis identified candidate variables (*p*-value ≤0.25) for the multivariable analysis. Multicollinearity among independent variables was assessed using standard error, variance inflation factor (VIF), and tolerance. Adjusted odds ratios (AOR) were used to measure the strength of associated factors, with a *p*-value <0.05 considered statistically significant. The Hosmer-Lemeshow statistic test was utilized to assess the fitness of the model.

## Results

### Socio-demography characteristics of participants

In this study, 612 adult respondents participated, making a 97.8% response rate. The mean age of the participants were 39 ± 0.55 standard deviation (SD) years. Considering sex, more than half, 367 (60%) of the participants were females. Among participants, about 421(68.8%) of the study participants were urban residents (Table 1).

**Table 1:** Socio-demographic characteristics of ART clients in public health facilities of Bahir Dar city administration, Northwest Ethiopia, 2020 (*n* = 612).

Variables	Frequency	Percent %
Age group in years		
18–28	132	21.6
29–38	194	31.7
39–48	159	26.0
> = 49	127	20.7
Marital status		
Single	143	23.4
Married	315	51.5
Divorced	83	13.6
Widowed	71	11.6
Religion		
Orthodox	541	88.4
Muslim	56	9.1
Protestant	11	1.8
Others	4	0.7
Ethnicity		
Amhara	573	93.6
Agew	15	2.5
Tigre	7	1.1
Oromo	10	1.6
Others	7	1.1
Educational status		
No formal education	290	47.4
Primary school	159	26.0
Secondary school	97	15.8
Diploma and above	66	10.8
Occupational status		
Governmental employee	86	14.1
Private employee	109	17.8
House wife	94	15.4
Farmer	82	13.4
Daily labor	98	16.0
Merchant	105	17.2
Other	38	6.2

### Behavioral characteristics of study participants

Of the participants, 427 (69.8%) individuals openly disclosed their HIV status. Additionally, 439 (71.7%) participants utilized reminder aids to take ART medication. In terms of experiencing stigma, 319 (52.1%) respondents reported facing it. Regarding awareness about ART, 420 (68.6%) participants had knowledge about it. Moreover, problematic alcohol use was reported by 103 (16.8%) respondents (Table 2).

**Table 2:** Behavioral characteristics of ART clients in public health facilities of Bahir Dar city administration, Northwest Ethiopia, 2020 (*n* = 612).

Variable	Frequency	Percent %
Feeling comfort taking ART in front of others		
Yes	203	33.2
No	409	66.8
Living condition		
living alone	234	38.2
living with others	378	61.8
Regular ART follow up		
Yes	474	77.5
No	138	22.5
Experienced stigma		
Yes	319	52.1
No	293	47.9
Receiving your ART		
own self	566	92.5
by other socials	46	7.5
Have you ever drunk alcohol?		
Yes	277	45.3
No	335	54.7
Problematic alcohol use (*n* = 277)		
Yes	103	37.18
No	174	62.82

### Clinical and health care system-related factors

The average treatment duration of the respondents were 59.6 ± 2 SD months. Among respondents, 32 (53.1%) had ≤350 CD_4_ counts at the base-line. The majority, 569 (93.0%) of respondents, were on a first-line ART regimen. Two hundred fifty-seven (42.0%) of the participants experienced high viral load. The mean time duration since HIV positive was 68 (SD ± 2.6) months. Five hundred seventy-eight (94.4%) participants had one pill daily and one hundred forty-one (23.0%) had additional medications other than ARVs. Two hundred twenty-four (36.6%) participants developed opportunistic infections (Table 3).

**Table 3:** Clinical and health care system related factors of ART clients in public health facilities of Bahir Dar city administration Northwest Ethiopia, 2020 (*n* = 612).

Variables	Frequency	Percent %
Duration on ART		
<1 year	83	13.6
1–5 years	314	51.3
>5 years	215	35.1
Functional status		
Working	561	91.7
Ambulatory	47	7.7
Bed ridden	4	0.7
Side effect		
Yes	190	31.1
No	422	67.1
Time spends to arrive ART center		
<1 h	386	63.1
1–2 h	167	27.5
>2 h	59	9.6
Duration since HIV positive		
<1 year	66	10.8
1–5 years	317	51.8
6–10 years	125	20.4
>10 years	104	17.0
Improvement in ART		
Yes	562	91.8
No	50	8.2
History of hospitalization		
Yes	295	48.2
No	317	51.7

### COVID-19 related characteristics

Out of the participants, 160 individuals, accounting for 26%, did not attend ART follow-up appointments because of the COVID-19 pandemic. The most significant portion, comprising 348 participants or 57%, had their follow-up schedules altered due to the pandemic (Table 4).

**Table 4:** COVID-19 pandemic-related factors of ART clients in public health facilities of Bahir Dar city administration Northwest Ethiopia, 2020 (*n* = 612).

Variable	Frequency	Percent
Lack of social support by COVID-19		
Yes	157	25.7
No	455	74.3
Lack of income source due to COVID-19		
Yes	353	57.7
No	259	42.3
Missing appointment due to COVID-19		
Yes	160	26.1
No	452	73.9
The main reason for missing follow-up		
Transport cost or restriction	95	59.4
Health facility closed	13	8.1
Fear of pandemic	50	31.2
Others	2	1.3
Follow up schedule changed due to COVID-19		
Yes	348	56.9
No	264	43.1
Adherence counseling and information in the context of COVID-19		
Yes	430	70.3
No	182	29.7

### Adherence to antiretroviral therapy

Of the total 612 study participants, 468 had good adherence to AR. In this study, good adherence to antiretroviral therapy was 76.5% (95% CI: 72.9, 79.7).

### Factors associated with adherence to ART

The variables shown as an association at bi-variable analysis could not remain significant at multivariable analysis. Only marital status, regular ART follow-up, adherence counseling & support in the context of the COVID-19 pandemic, and knowledge about ART persist as significantly associated with adherence to ART in both bi-variable and multivariable logistic regression analysis.

Respondents with divorced marital status were 55% less likely to adhere to ART than participants with married marital status (AOR = 0.45, 95%CI: 0.22, 0.90).

The odds of good adherence were three times higher among respondents who had regular follow-ups for clinical appointments compared with those who did not have regular follow-ups (AOR = 3.01, 95%CI: 1.81, 5.01).

Participants who had adherence counseling and information in the context of the COVID-19 pandemic were 2.57 times more likely to adhere to ART as compared with those who had not (AOR =2.57, 95%CI: 1.63, 4.08).

The odds of good adherence were 1.81 times higher among respondents with adequate knowledge than their counterparts (AOR = 1.81, 95%CI:1.11, 2.94) (Table 5).

**Table 5:** Bivariable and multivariable analysis between explanatory and adherence to ART among HIV-positive adults attending care and treatment in public health facilities of Bahir Dar city administration, Northwest Ethiopia, 2020 (*n* = 612).

Variables	Adherence		COR (95% CI)	AOR (95% CI)
	Good	Poor		
	No. (%)	No. (%)		
Marital status				
Single	112 (78.3)	31 (21.7)	0.90 (0.53,1.41)	0.91 (0.51,1.66)
Married	254 (80.6)	61 (19.4)	1	1
Divorced	46 (55.4)	37 (44.6)	0.30 (0.18,0.50)	0.45 (0.22,0.90)*
Widowed	56 (78.9)	15 (21.1)	0.90 (0.50,1.70)	0.81 (0.37,1.74)
Disclosure status				
Yes	339 (79.4)	88 (20.6)	1.67 (1.13,2.50)	1.3 (0.68, 1.87)
No	129 (69.7)	56 (30.3)	1	1
Living condition				
Living alone	166 (70.9)	68 (29.1)	1	1
Living with others	302 (79.9)	76 (20.1)	1.63 (1.11,2.37)	1.15 (0.68,1.95)
Regular follow up				
Yes	400 (84.4)	74 (15.6)	5.56 (3.67, 8.43)	3.01 (1.81, 5.01)^*^
No	68 (49.3)	70 (50.7)	1	1
Base line CD_4_				
<= 350	232 (71.4)	93 (28.6)	1	1
>350	236 (82.2)	51 (17.8)	1.85 (1.26,2.73)	1.49 (0.94,2.37)
Opportunistic infection				
Yes	157 (70.1)	67 (29.9)	1	1
No	311 (80.2)	77 (19.8)	1.72 (1.20,2.52)	1.06 (0.65,1.71)
Lack of social support due to COVID-19 pandemic				
Yes	114 (72.6)	43 (27.4)	1	1
No	354 (77.8)	101 (22.2)	1.32 (0.87,2.00)	0.82 (0.48,1.40)
Adherence counseling in the context of COVID-19 pandemic				
Yes	360 (83.7)	70 (16.3)	3.52 (2.38,5.21)	2.57 (1.63,4.08)^*^
No	108 (59.3)	74 (40.7)	1	1
Knowledge				
Knowledgeable	350 (83.3)	70 (16.7)	3.14 (2.13,4.62)	1.81 (1.11,2.94)^*^
Not Knowledgeable	118 (61.5)	74 (38.5)	1	1

*Significant, 1 reference category.

## Discussion

The primary objective of this study was to evaluate the influence of COVID-19 on adherence to ART and identify the factors associated with it. After conducting a multivariable analysis, it was determined that factors such as marital status, consistent ART follow-up, adherence counseling, support during the COVID-19 pandemic, and knowledge about antiretroviral therapy were significantly associated with adherence to ART.

The Good adherence to ART in this study was 76.5% (95% CI = 72.9,79.7). This aligns with previous research conducted in various locations, including China (72%) (7), Nekemt 77.9% (19), Jima Zone (73.6%) (20), Hara 71.8% (21), and Dubbo St. Marry Hospital, Areka Town, southern Ethiopia 78% (22). However, it surpasses the adherence rates reported in studies from Zimbabwe (65%) (23), Dre Dawa 65% (24), Jima university hospital 63.8% (25), and Nigst Eleni Mohammed memorial hospital 56% (15). The differences in adherence rates could be attributed to several factors, such as variations in study populations, timing of the studies, and specific healthcare settings. Socio-demographic characteristics, availability of healthcare services and support systems, and cultural factors could all influence ART adherence rates in these diverse settings. Additionally, differences in how adherence was assessed and what constitutes good adherence may have contributed to the variations observed across the studies.

The observed adherence rate of 76.5% in this study is lower than the rates reported in other studies conducted in different regions, including, Nepal 87.4% (26), India 89.5% (9), Indonesia 84.6% (27), Oromia regional state 98.8% (28), Hoshana 90.7% (11), Goba hospital 90.8% (29), Debre Birhan 95.5% (30), Gondar university specialized teaching hospital 88.2% (12), and Debre Markos referral hospital 88.6% (31). This disparity in adherence rates might be attributed to the specific study settings and contexts. Furthermore, the impact of the COVID-19 pandemic could be a contributing factor to lower adherence rates in this study. It’s possible that ART clients missed their routine HIV care appointments due to the pandemic, leading to interruptions in their ART medication. Additionally, the heightened focus on the COVID-19 pandemic may have diverted attention and resources away from HIV care and adherence, potentially affecting the results of this study.

Divorced clients exhibited reduced odds of achieving good adherence to ART compared to those who were married. This result is consistent with findings from studies conducted in Nigeria (32), Hoshana (11), and Nigst Eleni Mohammed memorial hospital (15). A possible potential explanation for this is that divorced individuals often face increased emotional and financial stress, which has been worsened by the uncertainties created by the COVID-19 pandemic. Due to the social isolation measures and disruptions in routine healthcare services, the stress levels of these individuals may further increase, making it challenging for them to prioritize and stick to their ART regimen. Moreover, divorced individuals may lack the social support systems that married individuals typically rely on during times of crisis, which could make it more difficult for them to cope with the pandemic’s challenges and maintain consistent adherence to ART.

Consistent attendance to ART follow-up appointments increased treatment adherence among HIV-positive adults compared to those who did not maintain regular clinical visits. This is comparable with studies done in Dre Dawa (24), Nigst Eleni Mohammed Memorial Hospital Hoshana (15), Nekemt Hospital (19), and Bale Goba Hospital (29). Maintaining regular attendance at ART follow-up appointments is critical for promoting treatment adherence among HIV-positive adults, which is supported by various studies. However, the COVID-19 pandemic has led to significant disruptions in healthcare services, which may hinder access to these appointments due to altered clinic schedules, transportation restrictions, and concerns about virus transmission. Despite these challenges, individuals who have continued to attend appointments have likely benefited from ongoing support and monitoring from healthcare providers. This continuity of care may have facilitated the timely identification and management of treatment-related issues, reinforcing adherence behaviors. Therefore, prioritizing regular clinical visits remains essential for promoting treatment adherence and enhancing health outcomes among HIV-positive individuals, even amid pandemic-related challenges.

The likelihood of achieving good adherence to ART was greater among individuals who received adherence counseling and information during the COVID-19 pandemic than those who did not receive such support. The finding is supported by the Federal Ministry of Health Ethiopia’s interim guidance for the provision of HIV services in the context of the COVID-19 pandemic in Ethiopia (13). A possible explanation for this observation is that providing up-to-date information and adherence support can enhance clients’ awareness and knowledge about their antiretroviral therapy and the ongoing pandemic, thereby contributing to better adherence.

Participants who possessed knowledge about ART were more inclined to adhere to their treatment than those who lacked such knowledge. The finding agreed with the studies conducted at Gondar University Specialized Teaching Hospital (12) and Jima Zone (20). One possible explanation for this trend is that clients with good knowledge are better equipped to comprehend the significance of taking their prescribed medication as directed. Moreover, they have a deeper understanding of the disease progression and its connection with COVID-19 in individuals with compromised immune systems.

### Limitations of the study

This study evaluated adherence via self-report, susceptible to social desirability and recall bias. However, participants were well-informed and encouraged to provide honest responses. This awareness likely minimized the effect of this limitation on the study’s findings’ credibility. Moreover, the study did not adequately address the impact of healthcare-related factors on treatment adherence.

## Conclusion

Compared to the standards set by the WHO and prior studies in Ethiopia, the level of adherence to antiretroviral therapy in this study was relatively low. According to the findings of this study, individuals who maintained regular ART follow-up appointments received adherence counseling and information specifically related to the COVID-19 pandemic and possessed knowledge about their treatment were more inclined to adhere to their medication regimen. On the other hand, People who identified as divorced had lower adherence to antiretroviral therapy.

### Implication

The findings of this study highlight the urgent need for improved efforts to increase adherence to ART among individuals living with HIV. Strategies that prioritize regular follow-up appointments, personalized counseling, and education on HIV treatment and emerging and reemerging health threats like COVID-19 could be beneficial in improving adherence rates.

Furthermore, the study suggests that social and emotional support systems may be crucial in medication adherence, as divorce was associated with lower adherence. Interventions aimed at enhancing support networks, providing psychosocial assistance, and addressing potential emotional distress among divorced individuals could potentially improve their adherence to antiretroviral therapy.

## Data availability statement

The raw data supporting the conclusions of this article will be made available by the authors, without undue reservation.

## Ethics statement

The studies involving humans were approved by Institutional Review Board of Bahir Dar University’s College of Medicine and Health Sciences. The studies were conducted in accordance with the local legislation and institutional requirements. The participants provided their written informed consent to participate in this study.

## Author contributions

CDG: Formal Analysis, Writing – review & editing, Writing – original draft. GWT: Writing – review & editing, Writing – original draft. BFS: Formal Analysis, Writing – review & editing, Writing – original draft.
